# The 2019 Coronavirus Disease (COVID-19): Possible Psychological Effects and Necessary Actions

**DOI:** 10.1017/dmp.2021.4

**Published:** 2021-01-08

**Authors:** Amir Hossein Goudarzian

**Affiliations:** Psychiatric Nursing, Student Research Committee, Mazandaran University of Medical Sciences, Sari, Iran

**Figure 1.**
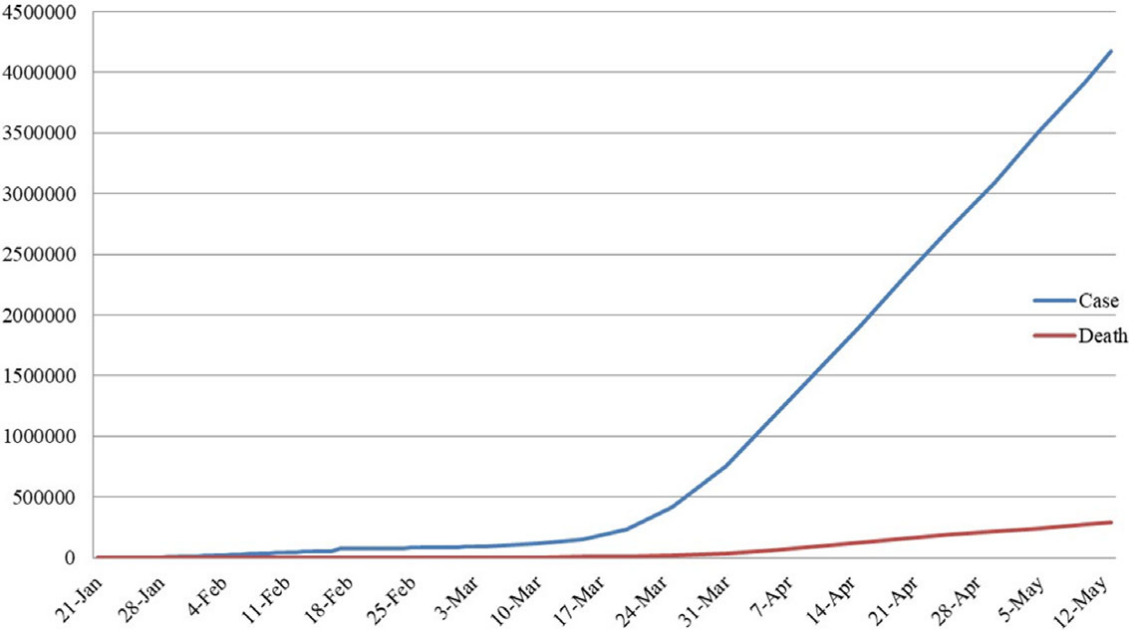
Total cases of disease and death caused by COVID-19 worldwide.

We are now fully aware that many of the pathological agents co-existing with us can also affect us psychologically. From these agents, viruses are the prime organisms associated with the stunning growth of psychological disorders.^1^ Unfortunately, coronavirus disease (COVID-19), whose virus originated in Wuhan, Hubei Province, China, in December 2019, has spread quickly to other regions and countries. The statistics presented in Figure 1 show the significant and rapid growth of COVID-19. The last reports from the World Health Organization show about 4 170 424 confirmed cases and 287 399 deaths until May 13, 2020 worldwide, as a result of the COVID-19 infection.^2^

As a result of these statistics, combined with the poor knowledge about this novel virus, psychological disorders have increased worldwide. We know from past experience that other types of coronaviruses have resulted in a high prevalence of psychological disorders. Bukhari et al.^3^ in 2016 and Kim et al.^4^ in 2018 warned that distress (fear and worry) and posttraumatic stress disorder (PTSD) were most prevalent in nurses dealing with the Middle East respiratory syndrome coronavirus (MERS-CoV). However, despite that, so far, the mortality rate of COVID-19 is significantly lower than that associated with other types of coronaviruses,^5^ other factors, mainly the significant outbreak and unknown development of this type of virus, may lead to great psychological damage. These psychological disorders, including distress, PTSD, and anxiety, may have negative effects on the health status of the population and increase susceptibility to COVID-19.^6^ Moreover, we need to increase the health status of medical workers, because they are on the battle front line. Indeed, Kang, et al.^7^ emphasized that many medical workers in Wuhan are prone to many types of psychological disorders because of high risk of infection, inadequate protection from contamination, overwork, frustration, discrimination, isolation, dealing with patients with negative emotions, lack of contact with their families, and exhaustion. Similar situations will occur in other countries as well if the virus is not contained.

So, what we can do? As a first step, all medical universities worldwide should hold special online workshops with emphasis on COVID-19 to better inform people about this virus and reduce the amount of associated distress. Then, supporting teams (including psychologists, psychiatrists, and psychiatric nurses) in each region must be formed to support mental health and screen for psychological disorders. Additionally, for medical workers, the governments should make an emergency decision to reduce work overload and increase personal protection. Actually, in Iran, some valuable strategies were implemented to reduce the workload of medical workers, including hiring a large number of nurses (in turn, reducing mandatory working hours), holding related online workshops for better learning about COVID-19, establishing encouragement payment for all medical workers fighting against COVID-19, and regularly monitoring medical workers for symptoms of COVID-19. Although contrary to expectations, now we can understand that, in many cases, complete quarantine can have bad results. In Iran, after the wide spread of COVID-19, all jobs were categorized in three levels (based on infection risk), and the vital jobs (those significantly affecting people’s lives) were opened with some special health safety measures in place. In fact, in the beginning of COVID-19 pandemic, psychological management is more important than other actions. These actions helped reduce psychological effects of COVID-19.

Finally, because understanding the psychological response of people is necessary for coping with any type of disaster, it is urgent that research on the subject be conducted worldwide, focusing on both the general population and specialized medical workers.
